# Supplementary material for: A generalized cortical activity pattern at internally generated mental context boundaries during unguided narrative recall
Source: eLife. 2022 May 30;11:e73693. doi: 10.7554/eLife.73693 (PMC9177147; doi:10.7554/eLife.73693)
Supplement: Transparent reporting form [file elife-73693-transrepform1.docx]

***eLife’s* transparent reporting form**

We encourage authors to provide detailed information *within their submission* to facilitate the interpretation and replication of experiments. Authors can upload supporting documentation to indicate the use of appropriate reporting guidelines for health-related research (see [EQUATOR Network](http://www.equator-network.org/%20)), life science research (see the [BioSharing Information Resource](https://biosharing.org/)), or the [ARRIVE guidelines](http://www.plosbiology.org/article/info:doi/10.1371/journal.pbio.1000412) for reporting work involving animal research. Where applicable, authors should refer to any relevant reporting standards documents in this form.

If you have any questions, please consult our Journal Policies and/or contact us: [editorial@elifesciences.org](mailto:editorial@elifesciences.org).

**Sample-size estimation**

- You should state whether an appropriate sample size was computed when the study was being designed
- You should state the statistical method of sample size computation and any required assumptions
- If no explicit power analysis was used, you should describe how you decided what sample (replicate) size (number) to use

Please outline where this information can be found within the submission (e.g., sections or figure legends), or explain why this information doesn’t apply to your submission:

Sample-size estimation does not apply to the current study because we reanalyzed an existing dataset collected for other purposes (Lee & Chen, 2021, bioRxiv). In Lee & Chen (2021), we recruited 21 participants for the fMRI experiment based on our prior study that showed robust event-specific neural patterns in the default mode network areas (22 participants recruited for Chen et al., 2017, Nature Neuroscience). The number of participants included in the current study can be found in the “Participants” subsection of the “Materials and Methods” section. We also cited the Lee & Chen (2021) study in the first paragraph of the “Materials and Methods” section.

**Replicates**

- You should report how often each experiment was performed
- You should include a definition of biological versus technical replication
- The data obtained should be provided and sufficient information should be provided to indicate the number of independent biological and/or technical replicates
- If you encountered any outliers, you should describe how these were handled
- Criteria for exclusion/inclusion of data should be clearly stated
- High-throughput sequence data should be uploaded before submission, with a private link for reviewers provided (these are available from both GEO and ArrayExpress)

Please outline where this information can be found within the submission (e.g., sections or figure legends), or explain why this information doesn’t apply to your submission:

Replication does not apply to the current study because the current study analyzed only one dataset from a single experiment. As for data exclusion, we excluded six participants due to excessive motion during fMRI scanning. This information is provided in the “Participants” subsection of the “Materials and Methods” section.

**Statistical reporting**

- Statistical analysis methods should be described and justified
- Raw data should be presented in figures whenever informative to do so (typically when N per group is less than 10)
- For each experiment, you should identify the statistical tests used, exact values of N, definitions of center, methods of multiple test correction, and dispersion and precision measures (e.g., mean, median, SD, SEM, confidence intervals; and, for the major substantive results, a measure of effect size (e.g., Pearson's r, Cohen's d)
- Report exact p-values wherever possible alongside the summary statistics and 95% confidence intervals. These should be reported for all key questions and not only when the p-value is less than 0.05.

Please outline where this information can be found within the submission (e.g., sections or figure legends), or explain why this information doesn’t apply to your submission:

The statistical tests used are described in detail throughout the “Materials and Methods” section. We visualized raw data from individual participants when we used bar graphs (Figure 3C, Figure 3-figure supplements 2C & 3, Figure 4, Figure 4-figure supplement 1 & 2A, Figure 5, Figure 5-figure supplement 2). We also visualized raw activation maps for individual participants in Figure 3B, Figure 3 supplement 1, and Figure 3 supplement 2B. The exact value of N, which was consistent across all analyses, is provided in the “Participants” subsection of the “Materials and Methods” section. We reported the methods of multiple comparisons correction (FDR correction following the Benjamini-Hochberg procedure for the whole-brain univariate analysis, and Bonferroni correction for all other analyses), dispersion and precision measures, Cohen’s d_z_, 95% confidence intervals, and exact p-values (if not smaller than .001) throughout the “Results and Discussion” section and all applicable figure captions.

(For large datasets, or papers with a very large number of statistical tests, you may upload a single table file with tests, Ns, etc., with reference to sections in the manuscript.)

**Group allocation**

- Indicate how samples were allocated into experimental groups (in the case of clinical studies, please specify allocation to treatment method); if randomization was used, please also state if restricted randomization was applied
- Indicate if masking was used during group allocation, data collection and/or data analysis

Please outline where this information can be found within the submission (e.g., sections or figure legends), or explain why this information doesn’t apply to your submission:

Group allocation does not apply to the current study because there was only one group/condition.

**Additional data files (“source data”)**

- We encourage you to upload relevant additional data files, such as numerical data that are represented as a graph in a figure, or as a summary table
- Where provided, these should be in the most useful format, and they can be uploaded as “Source data” files linked to a main figure or table
- Include model definition files including the full list of parameters used
- Include code used for data analysis (e.g., R, MatLab)
- Avoid stating that data files are “available upon request”

Please indicate the figures or tables for which source data files have been provided:

Source data files have been provided for Figure 1-figure supplement 3, Figure 3, Figure 3-figure supplements 2, 3, & 4, Figure 4, Figure 4-figure supplements 1 & 2, Figure 5, and Figure 5-figure supplements 1 & 2. The raw neuroimaging and behavioral data are publicly available via OpenNeuro (https://doi.org/10.18112/openneuro.ds004042.v1.0.0).
